# Lichens and Mosses as Biomonitors of Indoor Pollution

**DOI:** 10.3390/biology12091248

**Published:** 2023-09-18

**Authors:** Luca Paoli, Elena Bandoni, Luigi Sanità di Toppi

**Affiliations:** Department of Biology, University of Pisa, 56126 Pisa, Italy; e.bandoni2@studenti.unipi.it (E.B.); luigi.sanita@unipi.it (L.S.d.T.)

**Keywords:** biomonitoring, heavy metals, indoor air quality, indoor air pollution, lichen bags, moss bags

## Abstract

**Simple Summary:**

Human exposure to pollutants in indoor environments is a relevant health concern. Long-term monitoring data in indoor environments are largely missing due to a lack of adequate measuring devices. Biomonitoring (the use of living organisms to identify/assess potential hazardous exposure to chemicals and their effects) can provide useful information on indoor air quality and effects. Given their ability to intercept pollutants from the atmosphere, lichens and mosses are commonly used as outdoor biomonitors of atmospheric pollution by potentially toxic elements. Their application in indoor environment is recent but represents a promising output of the scientific research. In this review, indoor biomonitoring studies carried out using mosses and lichens have been compared, and critical issues and open matters have been underlined, as well as future perspectives related to their fruitful application in indoor environments. This review highlights the peculiarities of each study and the need for the development of shared harmonised protocols.

**Abstract:**

Biomonitoring in indoor environments is a recent application, and so far, indoor air quality (IAQ) has been investigated only in a few cases using photosynthesising biomonitors. On the whole, 22 studies have been selected and reviewed, being specifically focused on the assessment of IAQ using biomonitors, such as lichens (9 papers), mosses (10), or their combination (3). In general, indoor samples face an altered light regime, ventilation, and a reduced hydration, which should be taken into consideration during the design and implementation of indoor monitoring. This review highlights critical issues (and some solutions) related to sample devitalisation (moss), hydration during exposure, preparation of the exposure device (mostly lichen and moss bags), duration of the exposure, post-exposure treatments, assessment of the vitality of the samples, as well as data elaboration and interpretation. This review evidences the feasibility and usefulness of lichen/moss monitoring in indoor environments and the need to develop standardised protocols.

## 1. Introduction

Recent estimates showed that 92% of the world’s population (urban and rural) lives in places with air pollution levels exceeding WHO guidelines [1]. There is an increasing concern in monitoring the air quality of indoor environments, such as schools, dwellings, transports, shops, restaurants, offices, and working places in general, where most people spend more than 85% of their time [2]. Research on air quality has mostly focused on outdoor environments, whereas indoor air quality (IAQ) and its impacts on human health and well-being have received considerably less attention [3]. Research also highlighted the link between the quality of indoor environments and occupant well-being and comfort [4]. Despite a common belief that IAQ in urban areas is better than that outdoors, indoor concentrations of various chemicals and the consequent human exposures often exceed the corresponding outdoor values, significantly affecting the air we breathe [5,6]. Several compounds characterise IAQ and may originate from both outdoor as well as indoor sources. Among them, indoor VOCs, PAHs, NO_2_, CO, and heavy metals may seriously impact human health [7]. This is particularly true for sensitive categories of people, being chronically exposed to indoor pollution, such as babies, children, and people who are invalid or elderly [7].

Despite awareness concerning the problem of IAQ, long-term monitoring measurements in indoor environments are largely missing due to a lack of adequate measuring devices [8]. In fact, as Zechmeister et al. [8] clearly indicated, the most important sources of information about atmospheric indoor pollution are represented by instrumental measurements based on chemical–physical methods using stationary or mobile automatic gauges, which surely provide valuable information, but due to costs, their use is often time limited. In this sense, biomonitors can contribute to long-term monitoring in a more cost-effective way. Biological monitoring with lichens and mosses is of practical value in assessing exposure and risk caused by various pollutants, both outdoors and indoors. Lichens and mosses are poikilohydric organisms and have been widely used as biomonitors of outdoor air quality; however, so far, IAQ has been investigated only in a few cases using such organisms as biomonitors.

Since biomonitoring of IAQ is a fairly recent application, several matters still must be addressed to adapt the (outdoor) biomonitoring technique to indoor conditions. In this review, selected papers involved in indoor biomonitoring using mosses and lichens have been compared, and critical issues and open matters have been underlined, as well as future perspectives related to the use of biomonitors in indoor environments.

## 2. Materials and Methods

A systematic review was carried out with English terms in WOS, Scopus, as well as Google Scholar and finally integrated using a free search. The catch terms “indoor(s)”, “air quality”, “IAQ”, “air pollution”, “biomonitoring”, “heavy metals”, and “mosses” or “lichens” were combined to select suitable papers.

After duplicate removal, title screening, and abstract revision, the single papers were classified by type of biomonitor and ordered according to the year of publication. Only papers with full text in English have been included. Twenty-two studies have been selected and deeply examined, being specifically focused on monitoring IAQ using lichens (nine papers) and mosses (ten) alone, or in combination (three). With one exception (Ciani et al. [9]—manuscript under review during the preparation of this work, details gathered directly from the Authors), “grey literature” (i.e., technical reports, academic theses, and abstracts) was not considered. Likewise, studies based only on observations not supported by chemical measurements of the pollutants have not been included. Semi-confined spaces, such as parking garages (when encompassing underground environments, e.g., Vuković et al. [10]) and tunnels (Zechmeister et al. [11]), were treated as closed (hence, indoor) environments. Street canyons have not been considered as closed environments. This review allowed the identification of a cheating article on indoor biomonitoring [12]. For each selected paper, the following aspects have been highlighted: (1) topic investigated and geographic area; (2) lichen/moss species used as biomonitors; (3) duration of the exposure; (4) measured elements and/or parameters investigated; (5) protocols applied, including treatment of the samples prior, during, and after the exposure, as well as exposure conditions; and (6) main findings of the study.

## 3. Results

The main features of each study are summarised in Table 1 and Table 2. A detailed description is then reported in the following paragraphs, divided according to the biomonitor (lichen, moss, or a combination of both). For each study, specific information on the treatment of the samples (prior, during, and after the exposure) can be found in Table 3; details on the exposure devices and protocols, investigated elements (or other chemicals), analytical methods, and data treatment and interpretation are summarised in Table 4.

### 3.1. Topic and Geographic Area

Among the reviewed literature, the main research topics can be summarised as follows:

- IAQ in schools (Canha et al. [13,14,15]; Protano et al. [16]; Paoli et al. [17])—considering the most sensitive category (children) to indoor pollution, as well as the long time spent by children in indoor environments (at home or at school);
- IAQ in universities and in general working environments (Motyka et al. [18]; Demková et al. [19]; Ciani et al. [9]; da Silva et al. [20]);
- IAQ in houses/private environments (Al-Radady et al. [21]; Rajfur et al. [22]; Capozzi et al. [23]; Sorrentino et al. [24]; Paoli et al. [17]; Zechmeister et al. [8]);
- Problems related to traffic pollution, such as indoor contamination in parking garages (Vuković et al. [10]; Demková et al. [25]), tunnels (Zechmeister et al. [11]), car cabins (Paoli et al. [26]), and car workshops (Świsłowski et al. [27]);
- IAQ associated with cigarette smoke (Rajfur et al. [22]; Paoli et al. [26]);
- IAQ in a shooting range (Sujetovienė and Česynaitė [28]);
- Methodological aspects related to the monitoring devices (Al-Radady et al. [29]) or to the devitalisation of the samples (Motyka et al. [18]);
- Protection of cultural heritage (Winkler et al. [30]).

Twenty-one papers considered the content of major/trace elements in mosses/lichens (Table 2): some of them focused on a specific pollutant, such as Hg [9] or Pb [21], the latter much more relevant in the past than nowadays. Seven papers reported an assessment of the vitality of the biomonitor [13,14,17,20,26,27,28]. None of the indoor studies afforded, hitherto, the question whether lichens and mosses can actively contribute to purifying indoor air quality.

With the exception of the study by da Silva et al. [20] (in Brasil), the studies were carried out in Europe: mostly in Portugal [13,14,15], Central Europe [11,17,18,19,22,25,27], and Italy [9,16,23,24,26,30].

**Table 1:** Summary of the reviewed papers (L = lichen, M = moss).

	Authors (Year) [Reference]	Country	Topic	Exposure (In Weeks)	Species
L	Canha et al. (2012) [13]	Portugal	Schools	8	*Flavoparmelia caperata*
L	* Canha et al. (2014) [14]	Portugal	Schools	8	*F. caperata*
L	Protano et al. (2017) [16]	Italy	Schools	8	*Pseudevernia furfuracea*
L	* Canha et al. (2019) [15]	Portugal	Schools	8	*F. caperata*
L	Paoli et al. (2019) [17]	Slovakia	Schools, houses	8	*Evernia prunastri*
L	Paoli et al. (2019) [26]	Italy	Cars	9	*E. prunastri*
L	Sujetovienė and Česynaitė (2021) [28]	Lithuania	Shooting range	12	*E. prunastri, Ramalina farinacea*
L	da Silva et al. (2021) [20]	Brazil	University (lab)	3–12	*Cladonia verticillaris*
L	Winkler et al. (2022) [30]	Italy	Cultural heritage	12	*E. prunastri*
L+M	Demková et al. (2018) [25]	Slovakia	Parking	6	*Pleurozium* spp. (M), *Rhytidiadelphus* spp. (M), *Polytrichum* spp. (M), *P. furfuracea* (L)
L+M	Demková et al. (2019) [19]	Slovakia	University	4	*Dicranum scoparium* (M), *Hypogymnia physodes* (L)
L+M	Ciani et al. (2023) [9]	Italy	Herbarium	3 and 6	*P. furfuracea* (L), *Hypnum cupressiforme* (M)
M	Al-Radady et al. (1993) [29]	UK	Houses	4	*Sphagnum* sp.
M	Al-Radady et al. (1994) [21]	UK	Houses	4	*Sphagnum* sp.
M	Zechmeister et al. (2006) [11]	Austria	Tunnel	4	*Hylocomium splendens*
M	Motyka et al. (2013) [18]	Poland	Office	7	*H. splendens*
M	Vuković et al. (2014) [10]	Serbia	Parking	10	*Sphagnum girgensohni*
M	Rajfur et al. (2018) [22]	Poland	Houses	12	*Pleurozium schreberi*
M	Capozzi et al. (2019) [23]	Italy	Houses	12	*H. cupressiforme*
M	Zechmeister et al. (2020) [8]	Spain	Houses	8	*P. schreberi*
M	Sorrentino et al. (2021) [24]	Italy, Belgium	Houses	12	*H. cupressiforme*
M	Świsłowski et al. (2022) [27]	Poland	Car workshop	12	*Sphagnum fallax, P. schreberi, Dicranum polysetum*

* Based on Canha et al. (2012) [13].

**Table 2:** For each study: goal, experimental approach, and main findings. See text for details.

Authors (Year) [Reference]	Goal of the Study	Experimental Approach (How)	Main Findings
Canha et al. (2012) [13]	Assessing whether indoor exposure affects the vitality of lichen transplants and their capacity to detect pollution	By exposing lichens to indoors and outdoors in schools from urban and rural environments	Element accumulation in both outdoor and indoor environments; higher values of electric conductivity suggest physiological stress for indoor samples
* Canha et al. (2014) [14]	Characterising indoor and outdoor aspects of classrooms; identifying indoor sources of air pollution; charaterising electric conductivity of the thalli	By exposing lichens indoors and outdoors in schools from urban and rural environments	Traffic was identified as a source for As, Sb, and Zn; the use of chalk in classrooms was found as a source for indoor Ca; indoor electric conductivity was higher than outdoor-related values
Protano et al. (2017) [16]	Assessing the suitability of a fruticose lichen as an indoor biomonitor of trace elements and PAHs	By exposing lichens indoors and outdoors in schools from urban and rural environments	IAQ was only partially affected by outdoor pollutants in the investigated sites; *P. furfuracea* was deemed as suitable for indoor monitoring
* Canha et al. (2019) [15]	Characterising samples from the study by Canha et al. [13] with INAA using short irradiation	By exposing lichens indoors and outdoors in schools from urban and rural environments	Identification of other outdoor sources contributing to indoor depositions, in this case sea salt spray and industrial pollution
Paoli et al. (2019) [17]	Testing the contribution of air pollution to IAQ; comparing urban and rural areas; comparing the vitality of outdoor and indoor samples; testing the lichen *E. prunastri* to monitor IAQ	By exposing lichens indoors and outdoors in schools from urban and rural environments	Higher EC ratios in the urban environment; indoor accumulation for a few traffic-related elements (Cd, Cu, and Pb); IAQ not affected by outdoor conditions; the vitality of indoor exposed samples (chlorophyll *a* fluorescence) was not affected
Paoli et al. (2019) [26]	Assessing whether lichen transplanted in smokers’ cars accumulate nicotine and metal(loid)s from cigarette smoke and whether their vitality is affected	By exposing lichens inside a cabin of 5 smokers’ cars and 5 non-smokers’ cars	The effects of cigarette smoke can be detected using lichen transplants; the exposure to smoke alters lichen vitality (chlorophyll *a* fluorescence); indoor uptake also for Cu and Sb in non-smokers’ cars, caused by traffic
Sujetovienė and Česynaitė (2021) [28]	Evatuating trace elements and indoor thallus vitality	By exposing lichens at increasing distances from the firing line: 0, 5, and 10 m	Significant uptake of Pb detected in *E. prunastri*; altered chlorophyll *a* fluorescence emission; altered membrane integrity and oxidative stress in indoor exposed samples
da Silva et al. (2021) [20]	Biomonitoring of formaldehyde effects in indoor environments	By exposing lichens indoors in selected rooms at a university contaminated (and not) by formaldehyde and evaluating chlorophyll and phaeophytin contents	Indoor light (not uniform among the investigated environments) influenced chlorophyll content, so that, probably, a clear effect of the pollutant could not be detected
Winkler et al. (2022) [30]	Testing the use of lichen biomonitoring techniques for the preventive conservation of a historical building and its interiors	By exposing lichens along a mixed outdoor/indoor sampling transect at Villa Farnesina (Rome)	The magnetic/chemical properties of the transplants around and inside Villa Farnesina depended on the bioaccumulation of traffic-related particles (mainly Cu, Ba, and Sb); indoor contamination was limited/negligible
Demková et al. (2018) [25]	Comparing the indoor accumulation capacity of different moss and lichen taxa	By exposing lichens and mosses in an underground garage	Indoor uptake of traffic-related elements (RAFs > 1, including Fe, Mn, Ni, and Zn); accumulation varies according to the species; usefulness of combining of mosses and lichens
Demková et al. (2019) [19]	Assessing indoor air pollution in a university building; comparing two biomonitors; investigating the effect of sample hydration	By exposing lichens and mosses in various university environments and keeping half of the material hydrated	The hydration treatment (as carried out) did not influence the measured concentrations. Higher uptake of Cd and Mn in the moss and of Al, Cr, Cu, Fe, Ni, Pb, and Zn in the lichen. Labs were more contaminated than offices
Ciani et al. (2023) [9]	Evaluating the indoor residual contamination caused by mercury bichloride used in the past to protect herbarium specimens from insects	By exposing lichens and mosses in various rooms of the Herbarium at the University of Florence	Hg accumulated in all exposed biomonitors, suggesting indoor-air contamination from HgCl_2_ released by plant specimens
Al-Radady et al. (1993) [29]	Testing the efficacy of moss-bags as biomonitors of indoor pollution	A methodological study carried out using a series of experiments with mosses exposed indoors and outdoors	Keeping the moss constantly hydrated (with deionised water) improved its collection efficiency both indoors and outdoors
Al-Radady et al. (1994) [21]	Assessing indoor/outdoor Pb contamination	By exposing indoors and outdoors devitalised and irrigated moss bags	Peaks of Pb depositions (of outdoor origin) in proximity of the windows and a decrease within few meters inside the rooms
Zechmeister et al. (2006) [11]	Biomonitoring road traffic emissions in a tunnel	By exposing mosses (wooden frames) inside a tunnel and along five major roads	Mosses were potentially suitable as biomonitors in tunnels; concentrations were comparable to those derived from instrumental monitoring
Motyka et al. (2013) [18]	Biomonitoring indoor pollution and comparing irrigated (vital) with devitalised moss samples	By exposing three monitoring boxes (for hydrated samples) and three plastic bags (for devitalised ones) in an office, ca. 2 m above the floor	Hydrated samples showed higher Sb; Si; and to a lesser extent, Pb contents, while no difference appeared for Cu and Hg
Vuković et al. (2014) [10]	Biomonitoring indoor pollution by PM, heavy metals, and PAHs in parking garages	Concerning biomonitoring, by exposing moss bags next to the entrance, inside the garage (2.5 m above the floor)	The moss reflected small-scale variations in enclosed spaces: higher element concentrations in the vicinity of the entrances than in the interior.
Rajfur et al. (2018) [22]	Biomonitoring indoor pollution from tobacco smoke	By exposing indoor and outdoor moss bags (living gametophytes) in five kitchens (smoke) and five bedrooms (no smoke)	Mosses in smoking areas accumulated higher levels of metals than those exposed in non-smoking areas.
Capozzi et al. (2019) [23]	Testing moss bag efficacy to discriminate I/O elements and contributing to source apportionment	By exposing mosses in 12 coupled I/O sites in urban and rural areas in Campania (S Italy). Indoors in bedroom and living room, outdoors in balcony; 2 m from the floor	Moss bags distinguished between I and O sources. Traffic affected indoor pollution in urban areas; B, Mo, and Se were enriched outdoors; Ni, Cr, and V were enriched indoors
Zechmeister et al. (2020) [8]	Biomonitoring IAQ	By exposing mosses (wooden frames as in Zechmeister et al. [11]) indoors and outdoors in houses in the town of Girona	Concentrations of almost all elements increased both indoors and outdoors. Except for Cd, higher concentrations were found in outdoor mosses
Sorrentino et al. (2021) [24]	Investigating atmospheric metal pollution in 20 paired indoor–outdoor sites located in the urban areas of Naples (Italy) and Antwerp (Belgium)	By exposing moss bags in triplicate in bedrooms and living rooms (indoors) at 2 m above the floor and on the windows facing the street (outdoors)	Higher concentrations outdoors. Samples in Belgium enriched by elements of anthropic origin; in Italy by terrigenous elements. I/O ratios (mostly < 0.75) suggested that IAQ was strongly affected by outdoor conditions
Świsłowski et al. (2022) [27]	Assessing element accumulation and vitality (chlorophyll *a* fluorescence) of the samples	By exposing moss samples outdoors (road and under a roof) and indoors of a car workshop	Outdoor samples accumulated from wet and dry depositions (traffic and combustion processes); mosses exposed indoors (hence, not hydrated) had lost their vitality; most of the investigated elements had outdoor origin

* Based on Canha et al. (2012) [13].

### 3.2. Lichens

Canha et al. [13] carried out a transplant experiment in primary schools from urban (Lisbon) and rural areas of Portugal using the foliose lichen *Flavoparmelia caperata*. Samples were collected from a clean environment and exposed for two months (April–June 2010) indoors (classrooms) and outdoors (courtyards) of the studied primary schools. Lichens, once set to suitable bark pieces, were displayed inside trays and exposed in the classrooms, while those placed outside were bound to tree branches, in both cases at about 1.80 m from the floor. Accumulation data were interpreted in terms of exposed to control (EC) ratios [31]. Enrichment factors (EFs), accounting for element concentration in soil, were also evaluated. The vitality of the samples was determined based on membrane permeability measurements (electric conductivity). An accumulation (EC > 1.25) was found for several elements, both in outdoor and indoor environments. EFs pointed out a relevant accumulation of Sb and other elements in the urban area (reflecting traffic pollution) and, noteworthy, also in unexposed samples, suggesting a traffic source also in the rural area selected as a clean environment. An increased electric conductivity suggested the presence of physiological stress to indoor exposed samples.

The study by Canha et al. [14] represents an extension of previous work and highlighted the indoor origin of Ca deposition, likely from the chalk used on blackboards, while other contaminants, such as As, Sb, and Zn, were associated with anthropogenic sources, such as traffic. In addition, an assessment of the status of cell membranes (by measuring their permeability) suggested a possible stress to outdoor and indoor samples, especially in urban schools closer to the main roads.

The work by Canha et al. [15] consists of the assessment of the samples from previous studies with an instrumental neutron activation analysis using short irradiation (allowing the characterisation of Al, Cl, K, Mn, and V profiles). The results confirmed the resuspension of settled dust as a source for the concentrations recorded in *F. caperata* and according to the site and identified the contribution of sea salt spray and industrial pollution as further outdoor sources for indoor depositions.

Protano et al. [16] used the fruticose lichen *Pseudevernia furfuracea* as biomonitor of trace elements (As, Cd, Cr, Cu, Hg, Ni, and Pb) and polycyclic aromatic hydrocarbons (PAHs) in five selected primary schools in Latium (Central Italy): one in a highly urbanised area and four in rural settings. Lichen bags (each containing about 10 g of material) were exposed for two months (February–April 2014), and indoor (classrooms) and outdoor (gardens in the schools) environments were compared (EC ratios were used for data interpretation). Higher outdoor concentrations of trace elements and PAHs were found in the urban area (affected by traffic emissions), and EC ratios overall reflected a bioaccumulation for all investigated elements. On the other hand, indoor/outdoor ratios were <1, indicating that indoor air was only partially affected by outdoor pollutants, except for Cd in the urban area and for Hg and PAHs in the rural area. Traffic emissions were less relevant in the rural areas, and moreover, an explication for higher indoor PAHs could be that windows were generally closed during the winter season, especially in rural areas (the weather was colder than in the urban area).

During a citizen science experiment that involved teachers and students, Paoli et al. [17] assessed indoor air quality in the urban area of Bratislava and in a rural area (Madunice, Slovakia) using transplants of the lichen *Evernia prunastri*. Lichen bags were placed for two months indoors and outdoors in a school and a private house for each study area. Samples exposed indoors were regularly sprayed (not washed) with distilled water (up to three times per week). Lichens exposed outdoors significantly accumulated (EC ratio) most of the investigated elements (Al, As, Cd, Cr, Cu, Fe, Pb, S, Sb, V, and Zn) in the urban area, while only a few (As, Cd, Cu, Pb, and Sb) were accumulated in the rural area.

Independently of the outdoor concentrations, the indoor values were overall similar, both in rural and urban buildings. An indoor accumulation occurred for a few traffic-related elements (namely Cd, Cu, and Pb), but on the whole, IAQ in the schools was not affected by outdoor conditions. The vitality of indoor exposed samples (assessed by the analysis of chlorophyll *a* fluorescence emission) was not affected.

Paoli et al. [26] demonstrated that the effects of indoor pollution by cigarette smoke can be detected using lichen transplants. Lichen samples (*E. prunastri*) have been exposed for two months (between October and December 2017) inside the cabin of 10 volunteer’s cars (smokers and non-smokers): the bioaccumulation of heavy metals, nicotine content, and thallus vitality (by chlorophyll *a* fluorescence emission) have been investigated. Two different lichen bags have been placed within the cabin of each car (hanging from the rear-view mirror, or the lateral plastic handles). Prior to the exposure, the samples were washed via sequential immersions (three times) in deionised water, while during the exposure, the samples were not sprayed. After two months in smokers’ cars, lichens accumulated relevant amounts of metal(loid)s (Al, As, Cd, Cr, Cu, Ni, Pb, and Sb) and nicotine. The exposure decreased the photosynthetic activity of the thalli by 60% in comparison with non-smokers’ cars. Exposed to control ratios revealed an indoor uptake also for Cu and Sb in non-smoker’s cars, caused by traffic pollution.

Sujetovienė and Česynaitė [28] investigated indoor air pollution at a shooting range in Kaunas (Lithuania). Fruticose lichens (*E. prunastri* and *Ramalina farinacea*) were exposed using the lichen bag technique for 3 months. Ecophysiological parameters (potential quantum yield of primary photochemistry—F_V_/F_M_, electrical conductivity as indicator of cell membrane integrity, and TBARS as an indicator of oxidative stress) and the accumulation of metal(loid)s (Cd, Cu, Fe, Mn, Ni, Pb, Sb, and Zn) were measured. Since Pb bullets were used within the shooting range, an overall contamination from Pb was expected. In fact, based on their Figure 1, a significant uptake of Pb was detected in *E. prunastri*, in terms of exposed to control ratios (according to Frati et al. [31]), reflecting Pb dust released during shooting. A decrease in F_V_/F_M_ and a rise in oxidative stress (TBARS) were reported in both species, accompanied by a significant alteration of membrane permeability in *E. prunastri* (note that the comparison was carried out between indoor and outdoor exposed samples, used as a control for the ecophysiological parameters).

The study by da Silva et al. [20] focused on formaldehyde (CHOH), a toxic contaminant of indoor environments commonly used in the anatomy laboratory. The authors assessed the level of formaldehyde exposure to staff and students who attended a university anatomy lab and nearby indoor environments (Rio de Janeiro, Brazil). They also exposed lichen bags (containing the soil lichen *Cladonia verticillaris*) for 20, 40, 60, and 90 days (November 2018–January 2019) and recorded the content of photosynthetic pigments (as well as chlorophyll degradation) in the transplants. The variability in indoor light (not uniform among the investigated environments) appeared as a relevant driver for chlorophyll modifications, likely confounding the effects of CHOH contamination.

Winkler et al. [30] investigated magnetic properties and element depositions along a sampling transect at Villa Farnesina, Rome (Italy), a building regarded as one of the masterpieces of the Italian Renaissance. Lichen transplants (*E. prunastri*) were exposed for about 3 months (from October 2020 to the beginning of January 2021) at increasing distances from the closest road, highly concerned with particulate matter from vehicular traffic. An outdoor/indoor mixed sampling design was applied. The concentrations of Al, Ba, Cd, Cr, Cu, Fe, Ni, Sb, Sn, and Zn were investigated in the transplants, together with the magnetic properties. The magnetic properties of the transplants (inferred from magnetic susceptibility values, hysteresis loops, and first-order reversal curves) showed that the bioaccumulation of magnetite-like particles decreased exponentially with the distance from the road [30]. The exposure to traffic-related emissions for the indoor environment was very limited. The study witnessed the role of outdoor vegetation in intercepting traffic-originated particulate matter (especially Cu, Ba, and Sb from brake abrasions), hence protecting indoor cultural heritage and providing an essential conservation service. This field of application seems really promising for the future.

### 3.3. Mosses

At the beginning of the nineties, Al-Radady et al. [21,29] carried out pioneering research to test the efficacy of moss bags as biomonitors of indoor pollution (1993) and to assess the problem of Pb contamination inside the houses of the UK (1994).

In the first paper, they evaluated the feasibility of measuring metal deposition rates (Cu, Pb, and Zn) both indoors and outdoors using irrigated moss bags (*Sphagnum* spp.), comparing, respectively, devitalised (by HNO_3_) hydrated and dry mosses for 30 days. In fact, a common practice using moss bags is the preliminary devitalisation of the material, as also carried out in this research. They demonstrated that keeping the moss hydrated (with deionised water supplied by a simple device made by a capillary mat) improved its collection efficiency (of dry depositions), both indoors and outdoors. In the second study, the attention was focused on indoor pollution by Pb, selecting six houses in the areas of Bradford and Manchester (UK): besides measuring overall dust and Pb depositions outdoors and indoors (and in relation to the season), the study involved a transplant experiment with devitalised and irrigated moss bags (*Sphagnum* spp.), which were exposed for four weeks during 1990 (1–2 m from the floor in selected rooms). Noteworthy, the results highlighted peaks of depositions in proximity to the windows (in relation to wind flow) and a decrease within a few meters inside the investigated rooms, with wide variation according to the position of the room.

Zechmeister et al. [11] used the moss *Hylocomium splendens* to investigate emissions from road traffic within a tunnel in Wien (Austria). Moss samples were taken from a remote area in the Alps and exposed in September 2003 for four weeks by means of wooden frames (10 cm × 10 cm), covered with a thin plastic net with a mesh size of 1 cm × 1 cm. Mosses were not dried before the exposure and not sprayed during the exposure. Selected elements (mostly heavy metals) and PAHs were analysed using ICP-AES, AAS, and gas chromatography/mass spectrometry detection (GC-MSD). Enrichment factors were used for data interpretation. Concentrations were significantly higher in moss bags exposed within the tunnel (for all investigated substances) than along busy roads outside tunnels.

Motyka et al. [18] exposed *H. splendens* for 49 days within a working environment (office) in the Czech Republic. Samples were taken from a clean area; then, after washing in distilled water, half of the material was devitalised (24 h, 120 °C) and exposed at about 2 m from the floor, while the other half was not devitalised but kept hydrated during the exposure in a sampling device set up for this purpose. Trace elements (Cu, Hg, Pb, Sb, and Si) were evaluated on a weekly basis. Hydrated samples featured higher levels of Sb, Si, and to a lesser extent, Pb, while no difference appeared for Cu and Hg.

Vuković et al. [10] investigated indoor pollution by heavy metals in parking garages of Belgrade (Serbia) using moss bags (*Sphagnum girgensohnii*). Samples were collected in May 2011 from a remote area near Dubna, cleaned from extraneous material (not devitalised), and gently air-dried. Moss bags were prepared and exposed for 10 weeks during autumn. The study included a multi-pollutant assessment of air contaminants (PM_10_, trace elements, and PAHs) and an evaluation of health risks for employees. The results from the moss monitoring refer to 10 elements (Al, Ba, Cd, Cr, Cu, Fe, Ni, Pb, Sr, and Zn), whose values were interpreted in terms of absolute concentrations. The moss bags at the garage entrance accumulated higher amounts of metals than those exposed in the interior, likely in the form of dry particulate matter. Noteworthy, it was hypothesised that the lower relative element enrichment within the garages can be due to the dry indoor environment, which limited both moss physiological activity and further (ionic) element uptake.

Rajfur et al. [22] monitored indoor pollution from tobacco smoke using the moss *Pleurozium schreberi* in Poland. The study consisted of a comparison of samples (moss bags prepared with living material) exposed for three months inside kitchens (smoking areas) and bedrooms (non-smoking areas). Heavy metals associated with tobacco smoke were assessed (Cd, Cu, Fe, Hg, Mn, Ni, Pb, and Zn) with AAS, and noteworthy, hair samples from smokers and non-smokers were also analysed. Relative accumulation factors (RAFs) were used for data interpretation. Mosses exposed in smoking areas showed higher RAFs (all elements except Ni) than samples exposed in non-smoking areas. Some elements increased even in bedroom samples, suggesting the movement of pollutants to the supposed “clean” area. As expected, higher heavy metal concentrations were determined in hair samples from smokers.

Capozzi et al. [23] compared indoor/outdoor element deposition using the moss bag technique (*Hypnum cupressiforme*). For this purpose, they selected six rural and six urban sites in the area of Naples (Campania, Italy), where coupled (indoor/outdoor) exposure of samples was carried out for 12 weeks (May–July 2017). Indoor samples were exposed in bedrooms or living rooms (2 m above the floor), while outdoor samples were exposed on the respective balconies (not protected from wind and rain).

For data evaluation, “elements were considered enriched when in 60% of the sites, post-exposure concentration exceeded pre-exposure concentration plus two folds the standard deviation” [23]. Overall, the content of metal(loid)s was higher in outdoor exposed moss bags, and urban sites were more impacted than rural sites. A significant enrichment in several elements was found outdoors in the case of As, B, Ca, Co, Cr, Cu, Mn, Mo, Ni, Sb, Se, Sn, Sr, V, and Zn. On the other hand, a subset of elements (As, B, Cr, Mo, Ni, Se, and V) was enriched also in indoor samples. The use of indoor/outdoor ratios, allowed for contributing to source apportionment: Ni, Cr, and V were specifically enriched in most indoor samples, supporting the presence of indoor emitting sources.

Zechmeister et al. [8] investigated indoor and outdoor pollution in an urban area of Girona (Spain) by exposing moss samples (*P. schreberi*) for 8 weeks (May–July 2008) indoors and outdoors in 20 selected households. The sampling devices were similar to those described in Zechmeister et al. [11]. Outdoor samples were placed at a sheltered location (to prevent wet depositions), mostly on balconies. The following elements were measured before and after the exposure: Al, Cr, Cu, Zn, Sn, Cd, Pb, Mo, and Sb. The concentrations of almost all elements increased both indoors and outdoors. Except for Cd, higher concentrations were found in outdoor mosses than the corresponding indoor mosses.

Sorrentino et al. [24] investigated atmospheric metal pollution in 20 paired indoor–outdoor sites located in the urban areas of Naples (Italy) and Antwerp (Belgium). For this purpose, they exposed moss bags (devitalised *H. cupressiforme*) for 12 weeks (March–June 2019) in triplicate. Indoors, the bags were exposed in bedrooms or living rooms (2 m above the floor), while outdoors, the bags were exposed from the first to the third floor (as specified by the authors, fixed to a stick placed on the windows facing the street side, with no protection against rain or wind). Element concentrations were higher in the moss-bags exposed outdoors. The results revealed a similar accumulation profile, while some differences were related to the specific environments (e.g., Ag, As, Cd, Mo, Pb, and Sb in Belgium, with depositions enriched by elements of anthropic origin; Ca, Mg, Co, Cr, Sr, Ti, and U in Italy, with depositions enriched by terrigenous elements). The use of indoor/outdoor ratios (mostly lower than 0.75) suggested that the indoor pollution was strongly affected by outdoor conditions.

Świsłowski et al. [27] investigated indoor and outdoor pollution in and around a car workshop in Poland. They exposed three moss species (*Sphagnum fallax*, *P. schreberi*, and *Dicranum polysetum*) for 90 days (14 November 2020–14 February 2021) using the moss bags method. The content of 25 elements was measured, as well as chlorophyll *a* fluorescence emission (as an indicator of moss vitality). Relative accumulation factors (RAFs) were used for the interpretation of element concentrations. Most of the investigated elements originated from outdoor depositions; however, the results also suggested higher indoor uptake for Al, Ba, Cr, and Fe (despite it not being clear which is which, from their Figure 3).

### 3.4. Combination of Biomonitors

Demková et al. [25] compared the accumulation capacity of three mosses (*Pleurozium* spp., *Polytrichum* spp., and *Rhytidiadelphus* spp.) and the lichen *P. furfuracea* exposed in moss and lichen bags in an underground parking in the town of Prešov (Slovakia). Samples were collected in June 2016 from a remote area. Prior to the exposure, the material was cleaned from impurities, washed in distilled water, and then air-dried (60 °C for 24 h). The samples were exposed for 6 weeks in 2017 (the experiment was carried out twice, in May and October). Fourteen elements were investigated (Al, As, Ba, Cd, Co, Cr, Cu, Fe, Li, Mn, Ni, Pb, Sb, and Zn), and relative accumulation factors (RAFs) were used for data interpretation. A significant uptake was detected in the case of Fe, Mn, Ni, and Zn in the parking lot. The overall accumulation capacity followed the order *Pleurozium* spp. > *Rhytidiadelphus* spp. > *P. furfuracea* > *Polytrichum* spp., but since it changed depending on element and taxon, the study highlighted the usefulness of combining different biomonitors to obtain a clearer picture of indoor air pollution.

Demková et al. [19] assessed indoor air pollution in a university building (Prešov, Slovakia) and compared four room types (laboratories, halls, offices, and pc rooms) by exposing lichen (*Hypogymnia physodes*) and moss bags (*Dicranum scoparium*) for one month in 2017. Samples were collected in July 2017 from a remote area. Prior to the exposure, the material was cleaned from impurities, washed in distilled water, and then air-dried at room temperature. The study also included a comparison of hydration treatments: in each room, one batch of samples was sprayed (once a week); the other batch was left untreated. A group of elements with potential toxicological concern was investigated (As, Al, Cd, Cr, Cu, Fe, Mn, Ni, Pb, and Zn), and relative accumulation factors (RAFs) were used for data interpretation.

Under the experimental conditions, hydration of the samples did not influence the metal uptake. Higher concentrations were found in the moss, namely for Al, Cr, Cu, Fe, Ni, Pb, and Zn, while the lichen accumulated much more Cd and Mn. Laboratories were more contaminated (by As, Cr, and Mn) than offices; pc rooms showed the highest values of Cd and Cu.

A noteworthy example of biomonitoring applied to indoor environments is a pilot study carried out to evaluate the residual contamination caused by mercury bichloride (HgCl_2_) [9]. Such a compound, also known as a corrosive sublimate, was used in the past as an insecticide to protect herbarium specimens, which, as a consequence of the treatment, remained contaminated and, moreover, may still release Hg^0^ in the atmosphere posing a potential health risk for the employees of the herbaria—as shown, e.g., by Oyarzun et al. (2007) [32] in Spain. The lichen *P. furfuracea*, the moss *H. cupressiforme*, and bark pieces of *Pinus nigra* collected in a remote area were transplanted to the inside of selected rooms of the herbarium of the University of Florence (Italy) to assess Hg contamination after 3 and 6 weeks of exposure. The results revealed a significant accumulation in all biomonitors, with peaks of 0.656 μg/g in the lichen and 0.533 μg/g in the moss, suggesting indoor-air contamination [9].

## 4. Discussion

The assessment of IAQ in work and life environments is of paramount importance to estimate the total risk of exposure for humans and to identify relevant pollution sources [23]. Biomonitoring of IAQ is a fairly recent application, and several matters still must be addressed to adapt the (outdoor) biomonitoring techniques to the indoor conditions. For such reason, in the following section, the reviewed papers have been compared to highlight different approaches, critical issues, and open matters, as well as to examine and discuss future perspectives related to the use of biomonitors in indoor environments.

### 4.1. Which Species?

Accounting for the fact that the research by Canha et al. [13] led to the publication of three papers [13,14,15], there are twenty independent studies considered here and related to indoor air pollution monitoring (three of them based on lichens and mosses together). Overall, six lichen species and seven mosses (identified up to species level) have been used for indoor monitoring. In the case of lichens, the species mostly used have a fruticose (shrub-like) thallus: *E. prunastri* (four), *P. furfuracea* (three), *R. farinacea* (one), *H. physodes* (one), and the soil lichen *C. verticillaris* (one). The foliose lichen *F. caperata* (often used as outdoor biomonitor) has been used in Portugal (one). Fruticose species are in general easy to collect, transplant and prepare for the analyses, and the thallus has a wide surface/volume ratio, considered as well suited to intercepting ambient particles. This holds true also in indoor environments. However, which part of the thallus has to be taken for measuring elements in indoor monitoring has been explicitly reported only in Paoli et al. [17,26]. They indicated that the marginal parts of the laciniae (up to 2.5 cm from lobe tips in *E. prunastri*) were selected for the analysis and that this choice is foreseen by the protocols generally applied in the field of biomonitoring with lichens (at least for fruticose lichens). As a rule of thumb, the outermost portions of the thalli should be selected, being better exposed and, hence, able to intercept pollutants. In the case of mosses, the selection of apical segments (ca. 3–4 cm) excised from the shoots in *H. splendens* [18], that of green apices in *H. cupressiforme* [23], the green upper parts in *S. girgensohnii* [10], and living gametophytes (green shoots only) in *P. schreberi* [8,22] was specified.

The studies with mosses were chiefly carried out with *Pleurozium* spp. (mostly *P. schreberi*) (four), *Sphagnum* spp. (four), *H. cupressiforme* (three), *H. splendens*, and *Dicranum* spp. (two).

### 4.2. Prior to the Exposure: Living or Dead Material?

Concerning sample treatment, referring to general protocols available for bioaccumulation (outdoor) studies with lichens and mosses has been suggested, where such aspects have already been faced (e.g., [33,34,35,36]). In the reviewed papers, all lichen-based studies (including those with both biomonitors [9,19,25]) employed living samples, while in 5 out of 10 moss-based studies, the samples had been devitalised prior to the exposure (Table 3). Devitalisation occurred via HNO_3_ [21,29] or heating [18,23,24]. In the study by Motyka and colleagues [18], the purpose was a comparison between devitalised and non-devitalised samples. Devitalisation is not generally applied in lichen monitoring, though it is a common practice in moss monitoring (e.g., [37]), allowing a standardisation of procedures and reducing the influence of moss metabolism on element uptake. Studies carried out to standardise sampling protocols (see [38] and references therein) have shown that lichen samples exposed alive (after water washing) and mosses devitalised with different pre-treatments (oven drying and acid washing) accumulated comparable amounts of several trace elements and that, in the same exposure conditions, moss bags featured higher values than lichen bags [38]; furthermore, the element uptake increased during rainy periods (i.e., with hydration). However, mosses and lichens may take up elements not only as particles but also in ionic form, hence requiring also active metabolism. Since such organisms are not just passive sorbents/samplers, we should consider that living samples provide unique information also on the biological effects of IAQ, extending their potential as biomonitors. In fact, according to the definition of biomonitoring (use of living organisms), Świsłowski et al. [39], referring to mosses, suggested the exclusion of devitalisation as a pre-treatment, since it would make mosses only dead adsorbents of analytes. On the other hand, common pre-treatments include removal of adhering macroscopic particles, such as dust and soil [11]; litter [8]; and in general, dead parts or other species growing together with the selected material (Table 3). Such a step is followed by washing through sequential immersions in EDTA/deionised/(bi)distilled water and then drying (e.g., [17,18,23,24,26,27]), eventually followed by devitalisation. Noteworthy, in the study by Ciani et al. [9], samples were cleaned from impurities, then air-dried, and frozen before the exposure in herbarium. Freezing in that case was a precaution to protect the exposure environment (herbarium) from a potential external contamination (e.g., by small organisms in lichen and moss transplants).

In general, the time span between collection and exposure should be as short as possible. If the samples are not prepared within seven days, it is recommended to store them dry in a freezer (e.g., [33]). Accounting for the time between collection and exposure reported in the reviewed papers, in most cases, the samples were exposed in the study sites within few weeks, with exceptions being [10] (about 6 months later) and [25] (about one year).

**Table 3:** Summary of sample treatments (prior, during, and after the exposure); - not reported or no specific treatment.

Authors (Year) [Reference]	Pre-Treatment of the Samples	Treatment during the Exposure	Treatment after the Exposure
Canha et al. (2012) [13]	-	-	Cleaned from extraneous material; not washed
* Canha et al. (2014) [14]	-	-	Cleaned from extraneous material; not washed
Protano et al. (2017) [16]	-	-	Cleaned from extraneous material; not washed
* Canha et al. (2019) [15]	-	-	Cleaned from extraneous material; not washed
Paoli et al. (2019) [17]	Samples washed via sequential immersions (three times) in deionised water	Hydrated (gently sprayed) twice a week	Cleaned from extraneous material; not washed; stored in paper bags at about −18 °C until the analysis
Paoli et al. (2019) [26]	Samples washed via sequential immersions (three times) in deionised water	-	Cleaned from extraneous material; not washed; stored in paper bags at about −18 °C until the analysis
Sujetovienė and Česynaitė (2021) [28]	Cleaned from extraneous material	-	-
da Silva et al. (2021) [20]	Dried at room temperature and kept in paper bags until experiment	-	-
Winkler et al. (2022) [30]	Samples washed with deionised water. Extraneous particles such as moss and bark fragments were removed using plastic tweezers	Samples were sprayed with deionised water once per week to allow sufficient humidity for the thallus metabolism	Samples were air-dried and stored at −20 °C until magnetic and chemical analysis
Demková et al. (2018) [25]	Samples washed via sequential immersions (three times: 20, 15, and 10 min) in distilled water, then hand squeezed, and dry out (60 °C for 24 h)	-	-
Demková et al. (2019) [19]	Samples cleaned from impurities, washed in distilled water, and then air-dried at room temperature	For each exposure condition, half of the material was sprayed with water once a week	-
Ciani et al. (2023) [9]	Samples cleaned from impurities, then air-dried, and frozen before the exposure in herbarium	-	-
Al-Radady et al. (1993) [29]	Devitalisation (by HNO_3_) and then washing in pure water	Half of the material was hydrated with deionised water supplied by a capillary mat	-
Al-Radady et al. (1994) [21]	Devitalisation (by HNO_3_) and then washing in pure water	The samples remained hydrated with deionised water supplied by a capillary mat	-
Zechmeister et al. (2006) [11]	Mosses were cleaned from soil particles and brown dead parts were removed manually	-	-
Motyka et al. (2013) [18]	Washing in distilled water; half of the material was devitalised (24 h, 120 °C)	Half of the material (that non-devitalised) was kept hydrated with deionised water supplied by a capillary mat	-
Vuković et al. (2014) [10]	Samples were not devitalised; the green upper part was separated and carefully cleaned from soil particles	-	-
Rajfur et al. (2018) [22]	Samples were not devitalised; green parts only were selected	-	Air-dried at room temperature
Capozzi et al. (2019) [23]	Washed with sequential elutions (EDTA, distilled, and bidistilled water) and then devitalised by heating (treatment according to Capozzi et al. [40])	-	Air-dried at room temperature
Zechmeister et al. (2020) [8]	The moss was cleaned from litter and adhering macroscopic particles. Samples collected and prepared according to ICP vegetation guidelines [36]	-	-
Sorrentino et al. (2021) [24]	Washed with sequential elutions (EDTA, distilled, and bidistilled water) and then devitalised by heating (treatment according to Capozzi et al. [40])	-	Air-dried at room temperature
Świsłowski et al. (2022) [27]	Samples washed with mineralised water. Samples collected and prepared according to ICP vegetation guidelines [36]	-	-

* Based on Canha et al. (2012) [13].

### 4.3. How to Expose the Samples? The Exposure Devices

There is a need for standardised procedures in biomonitoring indoor environments. The highest variability is probably the position and treatment of the transplants: Vuković et al. [10] mentioned the necessity to obtain information about the position of the moss bags in relation to the distance from ground/floor in indoor environments. Sorrentino et al. [24] highlighted that “due to the recent application of the moss bags methodology in indoor environments, a dedicated research to find the best exposure time and conditions should be organized to provide useful information for a shared harmonized protocol”.

Indoor sampling devices have usually been exposed between 1 and 3 m (garages) from the ground/floor, mostly at 2 m. Referring to the available data (since not all the authors included such information), the exposure devices consisted of lichen and/or moss bags in 12 out of 22 papers, with 2–10 g of lichen/moss material, dimensions from 10 cm × 10 cm up to 20 cm × 20 cm, and mesh sizes of 0.5–1 cm for lichens and 0.2–1 cm for mosses. Canha et al. [13] exposed the samples on bark pieces (6 cm × 6 cm) displayed inside trays; Ciani et al. [9] exposed the samples on plastic boxes over a plastic net; Al-Radady [21,29] used irrigated moss bags, i.e., mosses exposed over a polystyrene box, which allowed hydration of the material by capillarity mats; Zechmeister et al. [8,11] placed their moss samples in wooden frames covered by a thin plastic net; and Motyka et al. [18] placed their moss samples over polypropylene boxes (full of distilled water with a capillary system for living samples). According to the type of study, the samples were bound to variable adequate supports in situ or in structures set up for the study’s purpose (Table 4). In a car experiment, lichen bags were hung from the rear-view mirror or the lateral plastic handles [26]. The position inside the rooms (houses, offices, schools, etc.) still remains an open matter.

**Table 4:** Summary of exposure devices and protocols, investigated elements (or other pollutants), analytical methods, and data interpretation.

Authors (Year) [Reference]	Protocols for Exposure	Elements or Other Chemicals	Analytical Method	Data Interpretation
Canha et al. (2012) [13]	Indoor samples: lichens on bark pieces (6 cm × 6 cm) displayed inside trays; outdoor samples: bound to tree branches; in both cases, at about 1.80 m from the floor/ground	As, Br, Ce, Co, Cr, Cs, Eu, Fe, Hg, K, La, Na, Rb, Sb, Sc, Se, Sm, Tb, Th, Yb, Zn	Instrumental neutron activation analysis (INAA)	Exposed to control (EC) ratios; enrichment factors (EFs) accounting for soil concentrations; indoor/outdoor (I/O) ratios
* Canha et al. (2014) [14]	Indoor samples: lichens on bark pieces (6 cm × 6 cm) displayed inside trays; outdoor samples: bound to tree branches; in both cases, at about 1.80 m from the floor/ground	As, Br, Ca, Ce, Co, Cr, Cs, Eu, Fe, Hf, K, La, Na, Rb, Sb, Sc, Sm, Sr, Ta, Th, Yb, Zn	INAA	EC ratios; EFs; I/O ratios
Protano et al. (2017) [16]	Lichen bags (20 cm × 20 cm bags, 1 cm mesh size); 2 m above ground level on adequate supports	As, Cd, Cr, Cu, Hg, Ni, Pb, and 12 selected PAHs	Atomic absorption spectrometry (AAS) for trace elements; gas chromatography–mass spectrometry (GC-MS) for PAHs	EC ratios; I/O ratios
* Canha et al. (2019) [15]	Indoor samples: lichens on bark pieces (6 cm × 6 cm) displayed inside trays; outdoor samples: bound to tree branches; in both cases, at about 1.80 m from the floor/ground	Al, Cl, K, Mn, and V	INAA using short irradiation	EC ratios; EFs; I/O ratios
Paoli et al. (2019) [17]	Lichen bags: composed of 3–5 thalli (4–5 cm long) placed within a plastic net (mesh size 0.8 cm); exposure: outdoors, to the branches of trees, and indoors (three bags per room), hanging from available supports (2 m from ground)	Al, As, Ca, Cd, Cr, Cu, Fe, Pb, S, Sb, V, Zn	ICP-MS	EC ratios; I/O ratios
Paoli et al. (2019) [26]	Lichen bags: each lichen transplant is composed of 3–5 thalli (generally 4–5 cm long), gently placed within a plastic net (mesh size 0.8 cm). Lichen bags hanging from the rear-view mirror or the lateral plastic handles	Al, As, Cd, Cr, Cu, Fe, Ni, Pb, Sb, V, Zn (and nicotine)	ICP-MS; high-performance liquid chromatography (HPLC) for nicotine	EC ratios
Sujetovienė and Česynaitė (2021) [28]	Lichen bags: mesh size 0.5 cm, exposed 2 m from the ground	Cd, Cu, Fe, Mn, Ni, Pb, Sb, Zn	Inductively coupled plasma optical emission spectroscopy (ICP-OES)	EC ratios
da Silva et al. (2021) [20]	Lichen bags: 2 g of fresh lichen placed in porous nylon bags; 12 bags at each sampling sitePassive samplers for measuring atmospheric formaldehyde	Formaldehyde (not in the transplants)	Formaldehyde (samplers for indoor air) by spectrofluorimetry	Determination of the effects of the exposure based on pigments concentration
Winkler et al. (2022) [30]	Lichen bags: of homogeneous size, using a plastic net loosely bound and closed at the extremities. Outdoor samples were tied to tree branches at least 2 m from ground; indoors, they were tied to the velvet ropes behind the frescoed walls at ca 50 cm from the floor. At each site, three lichen bags were exposed.	Al, Ba, Cd, Cr, Cu, Fe, Ni, Sb, Sn, Zn	ICP-MS; see [30] for magnetic properties	Absolute concentration and deposition rates. Correlation with the magnetic properties of the exposed samples
Demková et al. (2018) [25]	Moss and lichen bags: 2 bags of each taxa (5 g of sample wrapped into the nylon net 10 cm × 10 cm) were placed in 10 sampling points indoor an underground garage, next to the entrance/exit, 3 m above the floor	Al, As, Ba, Cd, Co, Cr, Cu, Fe, Li, Mn, Ni, Pb, Sb, Zn	ICP-OES	Relative accumulation factors (RAFs)
Demková et al. (2019) [19]	Moss and lichen bags: 2 g of sample into nylon nets (15 cm × 15 cm)	As, Al., Cd, Cr, Cu, Fe, Mn, Ni, Pb, Zn	ICP-OES	RAFs
Ciani et al. (2023) [9]	Moss and lichen samples exposed in plastic boxes over a plastic net	Hg	DMA-80—Direct mercury analyser	Absolute concentration; accumulation (%) normalised to the duration of the exposure
Al-Radady et al. (1993) [29]	Irrigated moss bags: mosses exposed over a polystyrene box, which allowed hydration of the material by capillarity mats	Cu, Pb, Zn	AAS	Absolute concentration
Al-Radady et al. (1994) [21]	Irrigated moss bags tested in Al-Radady et al. [29]	Pb	AAS	I/O ratios
Zechmeister et al. (2006) [11]	Moss samples exposed in wooden frames covered by a thin plastic net (mesh size 1 cm × 1 cm)	17 heavy metals; PAHs	Inductively coupled plasma atomic emission spectroscopy (ICP-AES), AAS and GC-MS	EFs
Motyka et al. (2013) [18]	Non-devitalised moss (capillary matting from polypropylene boxes full of distilled water). Devitalised moss (plastic bags made from LDPE net). Treatment of the material as suggested by Adamo et al. [37]	Cu, Pb, Sb, Si, Hg	AAS (Pb, Sb), ICP-AES (Cu, Si), and advanced mercury analyser (Hg)	Absolute concentration; comparison between irrigated and devitalised samples
Vuković et al. (2014) [10]	Moss bags: 3 g of moss material in 10 cm × 10 cm nylon net bags with 2 mm mesh size. 2.5 m above ground.	Major and trace elements (Al, Ba, Ca, Cd, Co, Cr, Cu, Fe, K, Mg, Mn, Na, Ni, Pb, Zn)	ICP-OES for heavy metals in the moss (GC-MSD for PAHs in samplers)	Absolute concentrations
Rajfur et al. (2018) [22]	Moss bags: 5 g of moss material	Heavy metals (Mn, Fe, Ni, Cu, Zn, Cd, Pb, Hg)	AAS	RAFs
Capozzi et al. (2019) [23]	Moss bags: the paper refers to the bags in Capozzi et al. [40], where three different types of bags (rounded, flat, and Mossphere) have been tested.	53 elements, including rare earth elements	ICP-MS	I/O ratios
Zechmeister et al. (2020) [8]	Moss shoots mounted on wooden frame equipped with a polypropylene net (mesh size 0.9 cm × 0.9 cm). For NO_2_ analysis, Palmes diffusion tubes were mounted next to moss samples	Metal(loid)s (Al, Cr, Cu, Zn, Sn, Cd, Pb, Mo, Sb) and NO_2_	ICP-sector field MS	Absolute concentrations, I/O ratios
Sorrentino et al. (2021) [24]	Moss bags, as reported in Capozzi et al. [40]	30 elements (Al, Ag, As, Be, Ca, Cd, Co, Cr, Cu, Fe, Hg, K, Mg, Mn, Mo, Na, Ni, Pb, Pd, Rb, Rh, Sb, Se, Si, Sr, Ti, Tl, U, V, Zn)	High resolution ICP-MS for chemical analysis, saturation isothermal remnant magnetisation (SIRM) for magnetic analysis	I/O ratios
Świsłowski et al. (2022) [27]	Moss bag technique: samples were hung at about 2 m from the ground	Al, As, Ba, Br, Ca, Cl, Co, Cr, Cs, Fe, Hf, I, K, La, Mg, Mn, Mo, Na, Rb, Sb, Sc, Se, Sm, Sr, Ta, Th, U, V, Zn	INAA	RAFs

* Based on Canha et al. (2012) [13].

### 4.4. Duration of the Exposure and Assessment of the Vitality of the Samples

The time of exposure in indoor environments ranged between 3 and 12 weeks (Table 1). Most of the reviewed studies with mosses [10,22,23,24,27] adopted a time span of 10–12 weeks, while in the case of lichens, 8–9 weeks were deemed as a suitable period for monitoring IAQ [13,16,17,26]. The suggested time span for a monitoring campaign with lichen transplants (outdoors) generally ranges between 4 and 12 weeks [33]. In the case of mosses, guidelines accounting for transplants (e.g., [34]) report a possible duration between 2 and 24 months. Comparing their results with other indoor studies, Sorrentino et al. [24] and Capozzi et al. [23] noticed that a period of 12 weeks could be more suitable for mosses to detect measurable concentrations and a clearer picture of indoor pollution, considering that indoor environments are generally characterised by lower pollution level compared to outdoors, and therefore, a longer exposure time could be more appropriate [24].

Beyond pollution, lichen and moss metabolism is basically related to water availability and light irradiance [41]. Indoor conditions might cause physiological stress to the samples, likely due to the modification of microclimatic conditions, mostly determined by altered light and water regimes. Hence, the assessment of the vitality of indoor exposed samples can be influenced either directly by the effects of pollution (that should be detected) and/or by the indoor conditions (that should be negligible, or at least quantifiable).

Paoli et al. [17] reported that the photosynthetic efficiency of indoor lichens (*E. prunastri*, after 8 weeks) in schools and houses was comparable to that of the nearby samples exposed outdoors. Hence, such a time span was considered adequate to detect a signal upon element uptake but avoiding a physiological alteration of the samples. A similar result was obtained when exposing *E. prunastri* inside non-smokers’ cars: the vitality was not statistically different from that of control samples, and relevant alterations in the photosynthetic activity were measured in samples that travelled in smokers’ cars, which were caused by smoke and not by the stay inside the car [26]. On the other hand, Świsłowski et al. [27] reported a loss of vitality (chlorophyll *a* fluorescence emission) in their mosses exposed indoors for 12 weeks (and not hydrated). Sujetovienė and Česynaitė [28] reported altered chlorophyll *a* fluorescence emission and membrane integrity as well as oxidative stress in indoor exposed samples, while Canha et al. [13,14] observed symptoms of stress in their *F. caperata* exposed in schools, measuring higher values of electric conductivity (i.e., alteration in membrane permeability) in some of their indoor samples. The interaction between pollutants and the indoor conditions was evident in the study by da Silva et al. [20]: indoor light (not uniform among the investigated rooms) influenced chlorophyll contents in addition to the presence of formaldehyde, so that, probably, a clear effect of the pollutant could not be detected. However, Canha et al. [13] indicated that the use of lichens as indoor biomonitors is feasible, despite the fact that higher physiological stress may occur in indoor environments (as a consequence of the indoor conditions itself or as pollution effects). A comprehensive design of a biomonitoring study should be able to discriminate between the effects of indoor pollution and the exposure conditions. Specific sets of samples should be dedicated to this purpose, especially using non-destructive tests, e.g., routine chlorophyll fluorescence assays, e.g., maximum quantum yield of primary photochemistry, F_V_/F_M_ [42], and/or chlorophyll content [39].

### 4.5. Treatments during and after the Exposure

With mosses and lichens being poikilohydric organisms, hydration of the thallus highly depends on water availability in the surrounding environment. This aspect is particularly relevant in an indoor environment. In most studies, living samples have been exposed and retrieved at the end of the transplant without further treatments (Table 3). Otherwise, they have been hydrated by periodic spraying with water (lichens), once [19,30] or twice a week [17], or irrigated continuously (mosses), being kept hydrated with deionised water supplied by a capillary mat ([21,29]; half of the samples in Motyka et al. [18]). The hydration of the samples seems particularly important for mosses to maintain their vitality when exposed indoors [27]. However, particular attention should be paid when (gently) spraying the samples with water, in order to allow only hydration and not the leaching of elements from the lichen or the moss.

Post-exposure treatments include drying; cleaning from extraneous materials (mandatory for outdoor samples); and eventually, storage of the material (e.g., [33]). It is generally recommended that after exposure, samples are dried, but not washed, to prevent the release of particles trapped on lichen/moss surface. Studies on lichens confirmed that the washing procedure at this stage can unpredictably alter the elemental composition of the thalli (e.g., [43]) and, hence, affect data homogeneity and quality. The reviewed papers generally did not report particular information on this step (Table 3): the samples were air-dried at room temperature [22,23,24]; cleaned from extraneous material, but not washed [13,14,15,16]; and cleaned and stored dry in a freezer until analysis [17,26,30]. The practice of freezing dry samples ensures that thalli remain healthy for later physiological measurements [44].

### 4.6. Data Processing and Interpretation

Data interpretation has been generally based on common procedures applied for outdoor biomonitoring with lichens and mosses. Concerning lichens, all studies but one [20] investigated major and/or trace elements in the transplants, which were in most cases assessed using the ratio between the concentration of each element after and before the exposure, the so-called exposed to control (EC) ratio (according to Frati et al. [31]). The EC ratio is based on the deviation from a normal condition; the latter was assumed to be ±25% from the ratio of 1 (a sort of buffer interval for normal oscillations of the concentrations). Alternative possibilities have been the direct use of absolute concentrations or that of enrichment factors (EFs), when soil data were also available. Four of the studies including mosses [19,22,25,27] used relative accumulation factors (RAFs) as a tool for data interpretation (Table 4). For each element, RAFs are calculated based on the concentration in the moss/lichen after the exposure subtracted by, and then divided by, the content before the exposure. In this case, RAFs > 0.5 suggest a slight enrichment for the investigated element and values > 1 suggest a significant enrichment ([45] and references therein]. Capozzi et al. [23] considered an element as enriched if its post-exposure concentration exceeded the pre-exposure one at least two folds the standard deviation in 60% of the sites/measures.

Recently, Cecconi et al. [46] proposed the use of exposed to unexposed (EU) ratios for outdoor lichen transplants, a variation of EC ratios, that also includes data variability (both in unexposed and exposed samples) when attributing a site to a specific class of accumulation. The scale consists of five percentile-based classes corresponding to increasing levels of bioaccumulation in transplanted lichen samples, namely, “Absence of”, “Low”, “Moderate”, “High”, and “Severe” bioaccumulation [46]. This approach could be adapted also to indoor monitoring.

In order to disentangle major and trace metal pollution sources between indoor and outdoor environments, indoor/outdoor ratios (I/O) were often used, allowing for establishing (or at least trying to) the outer or internal sources for the investigated elements [13,14,15,16,17,21,23,24]. I/O ratios with values > 1 are usually considered to represent indoor sources for the measured concentrations, while values < 1 should indicate an outdoor origin. Noteworthy, Sorrentino et al. [24] suggested keeping the interval 0.75–1.25 as a prudential buffer interval of data variability, similarly to the approach by Frati et al. [31] for EC ratios. In addition, they suggested the use of the correlations between outdoor and indoor concentrations: a correlation may reflect a common origin; without any correlation, if the I/O ratio is > 1.25, then an indoor source can be hypothesised [24].

## 5. Conclusions

This review of the existing literature pointed out the need for the development of standardised protocols also for indoor biomonitoring because, so far, IAQ has been investigated only in a few cases using mosses and lichens as biomonitors. Since mosses and lichens are not just passive sorbents, the importance of the vitality of the selected biomonitor has been highlighted, which should be considered as an advantage and not a limitation for the use of living organisms to detect air pollution effects. In general, indoor samples face an altered light regime, ventilation, and a reduced hydration, which should be taken into consideration when designing indoor monitoring studies.

As a summary, in the case of lichens, the species mostly used was the fruticose lichen *E. prunastri*, while the studies with mosses were chiefly carried out with *Sphagnum* spp., *Pleurozium* spp. (mostly *P. schreberi*), or *Hypnum cupressiforme*.

All lichen-based studies employed living samples, while devitalisation was a common practice in moss monitoring. The sample treatment (prior and after the exposure), the time span between collection and exposure, as well as the analytical methods should refer to general protocols available for bioaccumulation in outdoor studies with lichens and mosses, where such aspects have been already faced.

The correct position and the treatment of the transplants during their stay still remain open matters: indoor sampling devices have usually been exposed between 1 and 3 m from the ground/floor, mostly at 2 m; however, the position was arranged according to the peculiarities of each study and the availability of adequate support for the exposure. The exposure devices usually consisted of lichen and/or moss bags, with 2–10 g of lichen/moss material, dimensions from 10 cm × 10 cm up to 20 cm × 20 cm, and mesh sizes of 0.5–1 cm for lichens and 0.2–1 cm for mosses. Alternatively, wooden frames or bark pieces were used as exposure device.

The time of exposure in indoor environments usually ranged between 3 and 12 weeks: most of the studies with mosses adopted a time span of 10–12 weeks, while in the case of lichens, 8–9 weeks were deemed as a suitable period for monitoring IAQ.

During their stay in indoor environments, living samples were exposed and retrieved without further treatments; alternatively, they were hydrated by periodic spraying with water (lichens), once or twice a week, or irrigated continuously (mosses) with deionised water supplied by a capillary mat. Non-destructive tests (e.g., routine chlorophyll fluorescence assays) can be used to assess the vitality of the samples, discriminating between the effects of indoor pollution and those of exposure conditions.

Data interpretation has been generally based on common procedures applied for outdoor biomonitoring with lichens and mosses, which could be further developed, integrated, and adapted to indoor monitoring. In order to disentangle major and trace metal pollution sources between indoor and outdoor environments, I/O ratios are often used.

As a concluding remark, in spite of some critical issues, this review evidenced the feasibility and usefulness of lichen/moss monitoring in indoor environments.

## Data Availability

Not applicable.
